# A Physiological Basis for Nonheritable Antibiotic Resistance

**DOI:** 10.1128/mBio.00817-20

**Published:** 2020-06-16

**Authors:** Mauricio H. Pontes, Eduardo A. Groisman

**Affiliations:** a Department of Pathology and Laboratory of Medicine, Penn State College of Medicine, Hershey, Pennsylvania, USA; b Department of Microbiology and Immunology, Penn State College of Medicine, Hershey, Pennsylvania, USA; c Department of Microbial Pathogenesis, Yale School of Medicine, New Haven, Connecticut, USA; d Yale Microbial Sciences Institute, West Haven, Connecticut, USA; University of Texas Health Science Center at Houston

**Keywords:** antibiotic tolerance, growth feedback regulation, persister

## Abstract

Antibiotics constitute one of the cornerstones of modern medicine. However, individuals may succumb to a bacterial infection if a pathogen survives exposure to antibiotics. The ability of bacteria to survive bactericidal antibiotics results from genetic changes in the preexisting bacterial genome, from the acquisition of genes from other organisms, and from nonheritable phenomena that give rise to antibiotic tolerance. Nonheritable antibiotic tolerance can be exhibited by a large fraction of the bacterial population or by a small subpopulation referred to as persisters. Nonheritable resistance to antibiotics has been ascribed to the activity of toxins that are part of toxin-antitoxin modules, to the universal energy currency ATP, and to the signaling molecule guanosine (penta) tetraphosphate. However, these molecules are dispensable for nonheritable resistance to antibiotics in many organisms. By contrast, nutrient limitation, treatment with bacteriostatic antibiotics, or expression of genes that slow bacterial growth invariably promote nonheritable resistance. We posit that antibiotic persistence results from conditions promoting feedback inhibition among core cellular processes, resulting phenotypically in a slowdown or halt in bacterial growth.

## Abstract (precis)

Antibiotics constitute one of the cornerstones of modern medicine. However, individuals may succumb to a bacterial infection if a pathogen survives exposure to antibiotics. The ability of bacteria to survive bactericidal antibiotics results from genetic changes in the preexisting bacterial genome, from the acquisition of genes from other organisms, and from nonheritable phenomena that give rise to antibiotic tolerance. Nonheritable antibiotic tolerance can be exhibited by a large fraction of the bacterial population or by a small subpopulation referred to as persisters.

## INTRODUCTION

The use of antibiotics as chemotherapeutic agents is an historic milestone in human achievement. Antibiotics have been used to control bacterial infections, decreasing morbidity and mortality, extending the length and quality of human life, and promoting economic growth (1–3). Antibiotics target essential bacterial processes, such as DNA supercoiling, transcription, translation, and cell wall biosynthesis (3, 4). Antibiotics are bactericidal when they promote bacterial killing and bacteriostatic when they inhibit bacterial growth.

**FIG 1:**
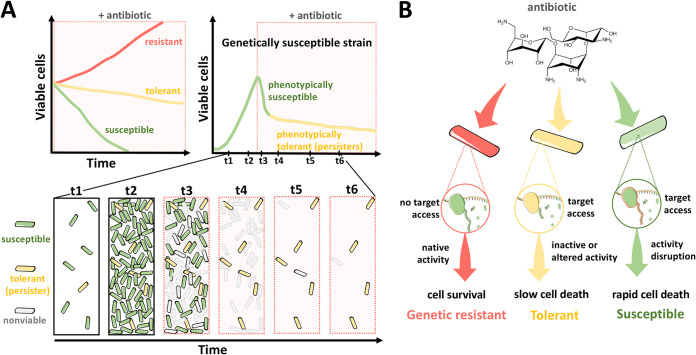
Effect of a bactericidal antibiotic on killing kinetics among susceptible, tolerant, and resistant bacteria. (A) Schematic representation of antibiotic killing during the population growth of susceptible, tolerant, and resistant bacteria (top left). Resistant cells (red line) are unaffected and are able to grow in the presence of the antibiotic. Tolerant cells (yellow line) are susceptible to the antibiotic but display slower killing kinetics than susceptible cells (green line). Schematic representation of antibiotic killing during the population growth of genetically susceptible bacteria (top right). Cells within this population can be partitioned into phenotypically susceptible cells, which comprise the majority of the population (green line), and a small fraction of phenotypically tolerant, persister cells (yellow line). Cartoon representation of antibiotic killing during the population growth of a genetically susceptible bacterium (bottom). Phenotypically susceptible (green) and phenotypically tolerant, persister cells (yellow) are depicted. (B) Cartoon schematics depicting the inferred action of an antibiotic (kanamycin) on susceptible, tolerant, and resistant cells. Ribosome cartoons were modified from Servier Medical Art, licensed under a Creative Common Attribution 3.0 Generic License.

Bacteria can survive and grow in the presence of bactericidal antibiotics through a variety of mechanisms. This antibiotic resistance can result from changes in genes encoding proteins that are targeted by antibiotics. Genetic changes may also result in an increased abundance of efflux pumps that decrease the cytoplasmic concentration of antibiotics below the threshold necessary for antibacterial action. In addition, bacteria can harbor products that destroy an antibiotic or that alter an antibiotic target so that it is no longer effectively inhibited by an antibiotic (Fig. 1). The latter products are often specified by genes in transmissible plasmids or transposable elements (4). Therefore, the acquisition of such genes can result in distantly related species harboring closely related antibiotic resistance determinants. The genes responsible for antibiotic resistance usually exhibit a limited phylogenetic distribution, most likely reflecting their association with species inhabiting niches with particular features that demand such a function and include recurrent antibiotic exposure (4, 5).

Genetically susceptible bacteria can also survive transient exposure to bactericidal antibiotics through a phenomenon known as tolerance. Tolerance is a behavior typically displayed by a large fraction of a bacterial population and results from genetic or nongenetic events. By contrast, persistence is a nongenetic form of tolerance exhibited by a small fraction of the bacterial population (Fig. 1A). Although the molecular basis for persistence is not well understood, such phenotypically resistant bacteria are thought to exist in a nonreplicative state in which the activity of their core biosynthetic machinery is transiently halted or diminished and, thus, protected from the lethal effects of antibiotics (Fig. 1B). In agreement with this notion, persister bacteria can withstand exposure to multiple antibiotics that target different core biochemical processes. In addition, when persisters are regrown in fresh media, they exhibit the same antibiotic susceptibility as the original culture (6–8). Unlike genetic resistance to antibiotics (4), nongenetic resistance has a broad phylogenetic distribution (8), suggesting that it is caused by a common attribute(s) central to diverse bacterial species and, perhaps, to all living organisms (9–11).

In this manuscript, we examine the physiological basis for nongenetic resistance to antibiotics. We posit that nongenetic antibiotic resistance results from a variety of mechanisms, all of which share the ability to dramatically decrease bacterial growth. A fundamental difference between genetic and nongenetic resistance to antibiotics is that the former relies on specific genes and pathways, whereas the latter does not. That eukaryotic organisms can display phenotypic resistance to a variety of drugs suggests that the underlying basis for nongenetic resistance to antimicrobial agents is conserved in evolution beyond bacteria (8–11).

## 

### The search for a genetic program responsible for nonheritable antibiotic resistance.

The clinical relevance of nonheritable resistance to antibiotics has led many laboratories to look for genetic determinants responsible for this phenomenon. Pioneering studies conducted with a laboratory strain of Escherichia coli established that mutations in the *hipA* gene (for high persistence) increased the frequency of persisters in the bacterial population >1,000-fold (12). Some of these mutations were later recovered in clinical isolates of E. coli and shown to confer increased antibiotic tolerance (13).

HipA is the toxin component of a toxin-antitoxin (TA) module that inhibits bacterial growth when not bound to the transcriptional regulator HipB (i.e., its cognate antitoxin) and the *hipAB* promoter (13). The toxin activity of HipA resides in its protein kinase domain, which phosphorylates the glutamyl aminoacyl tRNA synthase, thereby inhibiting protein synthesis (14, 15). Mutations in the *hipA* gene that result in high persistence map away from the regions specifying the kinase domain and the HipB binding region. These mutations decrease HipA’s ability to form homodimers, enabling monomeric HipA to promote persistence (13). The HipA/HipB pair is but one example of the many TA modules identified in bacteria (14–19). Despite the elegance of these and other studies that uncovered the biochemical activities of toxins and antitoxins (20, 21), as discussed below, TA modules are not necessary for tolerance/persistence to multiple antibiotics.

TA modules were first identified in plasmids as addiction modules that prevent plasmid loss by a mechanism referred to as postsegregation killing (see reference 22 for a recent review on TA modules). This mechanism involves an unstable antitoxin and a stable toxin that provokes the death of plasmid-free bacteria that fail to continuously produce the antitoxin. The vast majority of TA modules are encoded in bacterial chromosomes, and some species harbor dozens of TA modules (23), each consisting of a toxin that impairs cell growth and an antitoxin that inhibits the toxin (20, 21). However, the physiological role(s) that TA modules play in bacteria remains largely unclear (22).

Overexpression of toxins from TA modules can promote tolerance toward multiple antibiotics (8, 22, 24–27). For example, E. coli displayed tolerance to ciprofloxacin, ampicillin, and streptomycin when the *tisB* toxin gene was ectopically overexpressed from a plasmid (24). The *tisB* and *istR* genes form a TA module that is induced upon DNA damage by the bactericidal antibiotic ciprofloxacin. The TisB protein dissipates membrane potential, which decreases the concentration of ATP. This decrease is thought to increase tolerance to ciprofloxacin by preventing DNA synthesis. However, a different picture emerges when the *tisB* gene is expressed from its normal promoter and chromosomal location; partial tolerance is provided solely to its activating agent ciprofloxacin (24), thereby behaving similarly to an antibiotic resistance mechanism. Notably, *tisB* homologs are found only within selected members of the family *Enterobacteriaceae*, indicating that this TA module operates within restricted ecological contexts.

Tolerance toward multiple antibiotics can also be achieved upon overexpression of a variety of nontoxin genes (27–29). This is in spite of the fact that such genes encode proteins with different biochemical activities and subcellular locations and participate in a wide variety of cellular functions unrelated to those in which the toxins from TA modules act. These proteins include the chaperone DnaJ, the lipopolysaccharide-modifying PmrC, the transcriptional regulator Zur, the formate transporter FocA, the transhydrogenase PntA, and the sulfoquinovose isomerase YihS (27–29). The genes encoding these proteins share, along with toxin genes from TA modules, the ability to inhibit bacterial growth upon overexpression. Given that each of the proteins listed above inhibits growth by a different mechanism, we surmise that the multidrug tolerance phenotype associated with their overexpression results from the effects they have on cells aiming to maintain balanced growth.

TA modules were once believed to be required for antibiotic tolerance in several bacterial species. For example, the successive removal of TA module-encoding genes from the E. coli genome was reported to progressively decrease the level of persister organisms (30). However, that manuscript was later retracted (31), and the TA-mediated persistence originally reported was ascribed to contamination of the E. coli cultures with a bacteriophage (32). A different group ruled out a role for particular TA modules in E. coli tolerance by using bacterial cultures presumably free of the contaminating bacteriophage (33).

Surprisingly, 15-minute exposure of the facultative intracellular pathogen Salmonella enterica serovar Typhimurium (*S*. Typhimurium) to bone marrow-derived macrophages was reported to increase the frequency of nonreplicating persister bacteria >100-fold relative to *S*. Typhimurium grown in Luria Bertani (LB) broth (34). Such an increase was also described for LB-grown *S*. Typhimurium pre-exposed to pH 4.5, a pH slightly lower than that *S*. Typhimurium experiences inside a macrophage phagosome (34). In addition, *S*. Typhimurium mutants deleted for particular TA module genes, including *shpA/shpB* and *phD/doc*, were defective in persister formation (34). However, the acidic pH induction of persisters could not be recapitulated by another group that used the same *S*. Typhimurium strain and growth conditions (28). Moreover, a collection of engineered *S*. Typhimurium strains lacking individual TA modules, sets of TA modules, or all known TA modules retained wild-type antibiotic tolerance (28). Furthermore, deletion of the *shpA/shpB* and *phD/doc* genes in a different *S*. Typhimurium genetic background did not affect *S*. Typhimurium replication rate during systemic infection in mice (35), when bacteria reside and form persisters in macrophages (36–38).

In the Gram-positive bacterium Staphylococcus aureus, removal of the three known TA modules had no effect on persister levels of either exponentially growing or stationary-phase bacteria (39). Taken together with the findings obtained with the Gram-negative species E. coli and *S*. Typhimurium discussed above, these findings provide direct genetic evidence against TA modules being required for antibiotic tolerance.

### Reduced ATP abundance is associated with, but not required for, nonheritable antibiotic resistance.

The broad phylogenetic distribution of the persister phenotype (8) suggests that this phenomenon is implemented by a core, conserved feature of living cells and/or results from the action of a myriad of distinct cellular components that act on core cellular machines, impacting feedback control on such machines. The latter hypothesis finds experimental support from studies that link the defining property of persisters—high tolerance to multiple antibiotics—to a distinct cause or cellular perturbation. These perturbations range from mutations of particular genes to overexpression of various genes and also include exposure to chemicals or physical conditions that slow growth (12, 25–29, 39–45). For example, treatment of bacterial cultures with the ionophore *m*-chlorophenylhydrazone uncouples the electron transport chain from ATP synthesis, which results in ATP depletion, growth arrest, and multidrug tolerance (41).

Likewise, wild-type *S*. Typhimurium was 10,000 times more antibiotic tolerant when grown in defined media of low Mg^2+^ for 5 h than when grown in the same media for 2 h, possibly reflecting that ATP abundance was 15-fold lower at 5 h than at 2 h (28). Furthermore, expression of the soluble subunit of the ATP synthase resulted in a dramatic decrease in ATP abundance and a concomitant increase in *S*. Typhimurium survival in cefotaxime or ciprofloxacin (28). Cumulatively, these findings suggest that persister bacteria emerge as a consequence of a decrease in the cellular concentration of ATP (39, 42).

ATP is a universal chemical energy storage molecule utilized by all cells to fuel biosynthetic reactions, to transport solutes across biological membranes, and to synthesize proteins following its conversion into GTP. Therefore, changes in cellular ATP provide a unifying explanation for a decrease in the ATP concentration, driving tolerance across Gram-negative E. coli, Gram-positive S. aureus, and perhaps even eukaryotes. However, as detailed below, this notion fails to explain the root of the antibiotic persistence phenomenon.

First, at any given time, the cellular ATP concentration is determined by hundreds of anabolic and catabolic processes (46, 47). Therefore, fluctuations in ATP concentration must ultimately be explained in terms of changes in these processes. In agreement with this reasoning, correlations between low ATP concentrations and antibiotic tolerance have been attributed, in part, to the stochastic expression of the genes encoding tricarboxylic acid (TCA) cycle enzymes by a mechanism that remains unknown (48). Because the TCA cycle generates ATP (as well as other products), the abundance of TCA cycle enzymes would determine the ATP concentration in individual bacterial cells and, thus, antibiotic persistence.

Second, chemical treatments that result in ATP depletion promote multidrug tolerance to populations of E. coli and S. aureus (39, 41, 42). This observation is consistent with the hypothesis that low ATP concentration is the cause, or at least part, of a causal chain of events, leading to high, multiantibiotic tolerance. However, low ATP concentration is not necessary for tolerance to multiple bactericidal antibiotics. This is because treatment of wild-type *S*. Typhimurium with the bacteriostatic antibiotic chloramphenicol promoted tolerance despite dramatically increasing the ATP concentration (28). Chloramphenicol treatment increases the cellular ATP concentration because it is an inhibitor of translation, and translation is the most energy expensive activity cells undertake (49). Likewise, treatment of a laboratory strain of E. coli with tetracycline increased survival in ampicillin or ciprofloxacin >1,000-fold relative to bacteria that were not treated with tetracycline (41). Tetracycline is also a bacteriostatic protein synthesis inhibitor, but its mechanism of action is different from that of chloramphenicol (4). Because tetracycline and chloramphenicol inhibit bacterial growth, the antibiotic tolerance they promote can be ascribed to bacteria not growing.

Third, naturally emerging, phenotypically variant subpopulations of E. coli with rRNA promoter activity displayed tolerance to multiple antibiotics (42). Given that rRNA promoter activity increases with ATP concentration, bacteria exiting energy starvation conditions must have low ATP amounts (42). However, rRNA transcription increases not only when ATP concentration increases but also when growth rate increases. While the molecular basis responsible for the growth rate-dependent regulation of rRNA transcription is poorly understood, it is distinct from the control exerted by ATP abundance (50–55). Increases in growth rate lead to heightened rRNA transcription even though ATP amounts remain constant (53–55). In other words, the correlation between low rRNA transcription and subpopulations exhibiting multiantibiotic tolerance may reflect low ATP amounts, low growth rate, or both.

Fourth, subpopulations of S. aureus displaying multiantibiotic tolerance were presumed to have low ATP amounts by virtue of expressing genes normally induced at the stationary phase of growth and upon arsenate treatment (39). Because direct measurements of ATP amounts were not provided in this study and because the regulatory cascades and signals governing transcription of the tested genes are largely unknown, expression of the chosen reporter genes may reflect low ATP amounts, slow growth, or any other condition encountered during stationary phase or arsenate treatment.

Finally, when bacteria run out of ATP, they stop growing, which favors the formation of persisters. This in and of itself does not make low ATP concentration essential for persister formation because it is possible to achieve tolerance to multiple antibiotics by stopping growth while cells accumulate ATP to high concentrations.

### Depending on growth conditions, the signaling molecules ppGpp and pppGpp are required, dispensable, or detrimental for nonheritable antibiotic resistance.

The stringent response is a collection of molecular responses mediated by the production of GDP 3′-diphosphate (ppGpp) and GTP 3′-diphosphate (pppGpp) by bacterial cells experiencing nutritional starvation (56). ppGpp and pppGpp [from here on abbreviated as (p)ppGpp] control a wide variety of cellular functions and are implicated in antibiotic tolerance.

(p)ppGpp accumulation was first identified in E. coli experiencing amino acid starvation (57), and it is now known that (p)ppGpp can accumulate in bacteria starving for other nutrients, such as carbon and phosphate (58–62). When E. coli starves for an amino acid, the corresponding uncharged tRNAs can bind to the A site of ribosomes, and the stalled ribosomes are detected by the protein RelA, which is responsible for the synthesis of (p)ppGpp (63). E. coli also harbors SpoT, a protein with lower (p)ppGpp synthase capacity than RelA but which, unlike RelA, can also hydrolyze (p)ppGpp. Bacterial species differ in the number and classes of proteins that can make and break down (p)ppGpp (i.e., monofunctional versus bifunctional) (56).

(p)ppGpp exerts its regulatory function by binding a wide variety of proteins (64, 65). In E. coli, (p)ppGpp binding to RNA polymerase directly changes transcription of ∼15% of the genome (66). Two of the most salient changes are a decrease in the transcription of rRNA genes and an increase in transcription of amino acid biosynthetic genes. It makes intuitive sense for a bacterium experiencing amino acid limitation to decrease the synthesis of components of its protein synthesis machine and to increase the production of the enzymes responsible for making amino acids. Even in organisms experiencing plenty of amino acids (i.e., the substrates of protein synthesis) and ATP (i.e., the energy source to run protein synthesis), low cytoplasmic Mg^2+^ decreases rRNA synthesis in a (p)ppGpp-dependent manner (67). This is because Mg^2+^ is necessary for the assembly of functional ribosomes, and bacteria decrease the synthesis of rRNA when the cytoplasmic Mg^2+^ concentration drops below a certain threshold (67). As proteins are essential for cell growth, a decrease in the number of ribosomes decreases the rate of protein synthesis, which in turn reduces growth rate. We posit that such a reduction in growth rate can give rise to antibiotic tolerance.

A role of (p)ppGpp in antibiotic tolerance has emerged from both the recovery of clinical isolates bearing large amounts of (p)ppGpp and laboratory studies conducted with different bacterial species. For example, isolates of the Gram-positive bacteria S. aureus and Enterobacter faecium bearing large amounts of (p)ppGpp due to mutations in the genes responsible for (p)ppGpp synthesis (68, 69) caused persistent infections and required prolonged antibiotic treatment for clearing (56). In the laboratory, starving E. coli for certain amino acids increased resistance to the bactericidal antibiotic penicillin (70). Resistance appears to be mediated, in part, by the stringent response because the protective effect of amino acid starvation was exhibited at later times in a *relA* mutant (70). Moreover, deletion of the *relA* and *spoT* genes eliminated the persistence phenotype of an E. coli strain bearing the *hipA7* allele, which specifies a HipA variant that confers high persistence but is not toxic, unlike the wild-type HipA protein (71).

(p)ppGpp was proposed to promote antibiotic persistence in E. coli by increasing the fraction of free toxins from TA modules (72). According to this proposal, (p)ppGpp would inhibit the enzyme that degrades polyphosphate—termed exopolyphosphatase—resulting in the accumulation of polyphosphate, which in turn would activate the protease Lon to degrade the antitoxin from TA modules. The unbound toxins would then be free to inhibit their targets. In agreement with this notion, an E. coli mutant lacking the capacity to make (p)ppGpp due to a deletion of the *relA* and *spoT* genes was ∼100-fold less persistent to ciprofloxacin and ampicillin than the isogenic wild-type strain, and a *lon* null mutant was even less persistent than the *relA spoT* double mutant (72). However, the article describing these findings was later retracted (73).

In *S*. Typhimurium, a *relA spoT* double mutant and a *lon* mutant were reported to be ∼10 times and ∼100 times less tolerant to antibiotics than the wild-type strain, respectively, following bacterial internalization by macrophages (34). These phenotypes are reminiscent of those for E. coli in the retracted paper discussed above. In addition, the genes specifying 14 TA operons were reportedly upregulated 4- to 30-fold within 30 min of *S*. Typhimurium phagocytosis by macrophages (34). This upregulation was dependent on the *relA* and *spoT* genes and also observed upon acidification of the laboratory media, a condition that, as discussed above, was also reported to promote antibiotic persistence (34). By contrast, a group investigating the same *S*. Typhimurium strain and using the same experimental protocol found that the *relA spoT* double mutant retained wild-type tolerance to ciprofloxacin and cefotaxime in bacteria experiencing acidified LB medium (28). Moreover, (p)ppGpp was dispensable for the tolerance to cefotaxime displayed by an *S*. Typhimurium tryptophan auxotroph in medium lacking tryptophan (28).

Serine hydroxamate (Shx) is an amino acid analog often used to trigger the stringent response because it hinders the charging of seryl-tRNA with serine (74). It has been reported that Shx treatment of stationary-phase cultures of *S*. Typhimurium increased persistence ∼5-fold relative to bacteria grown in LB broth (34). This observation is puzzling because bacteria in stationary phase are already nutrient starved, which results in large amounts of (p)ppGpp (55, 61, 62, 75). This is why a different group testing the same *S*. Typhimurium strain established that Shx had no effect on tolerance when added to bacteria in the stationary phase but did render bacteria immune to cefotaxime when added to logarithmically growing bacteria (28). These sets of data argue that (p)ppGpp is dispensable for antibiotic tolerance elicited by a variety of conditions.

Actually, depending on the experimental conditions, the ability to make (p)ppGpp can hinder antibiotic tolerance. That is, a *relA spoT* double mutant survived cefotaxime treatment better than wild-type *S*. Typhimurium during growth in defined medium lacking amino acids (28). These results reflect that a *relA spoT* double mutant displays multiple auxotrophies and, thus, cannot grow in the absence of amino acids, whereas the wild-type strain can, rendering the latter, but not the former, susceptible to killing by bactericidal antibiotics (28, 62). In other words, the (p)ppGpp-promoted growth occurring in the absence of certain nutrients is what hinders tolerance in a wild-type strain but not in the (p)ppGpp-lacking *relA spoT* double mutant.

### Cells coordinate the activities of their core machineries.

Living cells are composed of defined sets of components. These components are dynamic in nature. Therefore, the synthesis, degradation, and recycling of these components allow cells to maintain homeostasis despite changes in their surroundings. While the overall chemical composition of cells varies across cell types and growth conditions (50, 76, 77), all cells share a set of components that participate in processes responsible for growth and viability, namely, the machineries carrying out the three core cellular processes—DNA replication, transcription, and translation.

**FIG 2:**
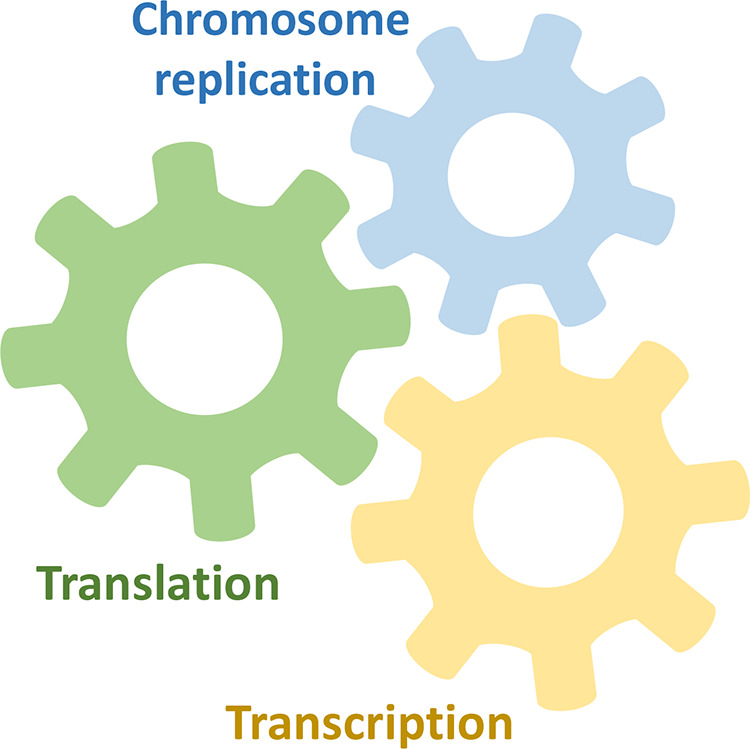
Cartoon depicting the three core cellular processes (chromosome replication, transcription, and translation) and coregulatory relationships among them.

In bacteria, the three core cellular processes are coregulated (Fig. 2). Early studies established that global transcriptional and translational activities control chromosome replication (78–81). Similarly, the translational status of a cell conveys information to the transcriptional and DNA-replicating machineries, modulating their activities (51, 61–65, 82, 83). That ribosome activity is required for the initiation of chromosome replication is conserved in distantly related bacterial species, such as E. coli and Bacillus subtilis (78–83). Other vital cellular processes are also coordinated, namely cell division that is regulated by the DNA replication status (see reference 84 for a recent review on this topic). Given that imbalances in cellular components can hinder growth or lead to a loss of cellular viability (62, 67, 82, 83, 85–101), feedback regulatory mechanisms ensure that cellular constituents are synthesized within a stoichiometric range compatible with life. That is, the activities of cellular components are coordinately regulated to avoid deleterious effects resulting from inefficient resource allocation, which can lead to the accumulation of toxic metabolites, the depletion of vital biosynthetic precursors, stoichiometric imbalances of cellular components, and the establishment of artificial, inhibitory molecular interactions that disrupt cellular processes. This fundamental property can explain noninheritable antibiotic resistance, a phenomenon that, as discussed above, is conserved across distantly related bacterial species.

In the laboratory, bacteria are typically grown under conditions favoring high catabolism, whereby ATP production from available substrates exceeds the energetic requirements of the cell. Under such conditions, bacteria dissipate excess ATP, and their growth rate is dependent on the availability of critical precursors rather than the energy to power nutrient transport and biosynthetic reactions (46, 47). Hence, under laboratory conditions, ATP amounts remain constant even when the growth rate is increased by nutritional up-shifts, which makes limiting precursors readily available, or by temperature increases, which speeds up biosynthetic reactions (46, 47, 53–55, 76, 102, 103). In other words, under typical laboratory conditions, ATP amounts are usually uncoupled from biosynthetic reactions, both of which are required for growth and targeted by antibiotics.

In addition to the intrinsic coregulation of DNA replication, transcription, and translation, ancillary proteins can also exert feedback control of the core machinery in response to environmental cues. For instance, the *mgtC* gene, which was acquired by horizontal gene transfer in *S*. Typhimurium (104), specifies a protein that reduces ATP concentration, thereby decreasing the synthesis of rRNA (67). MgtC is required when the *S*. Typhimurium cytoplasmic Mg^2+^ concentration drops below the threshold that compromises the assembly of functional ribosomes (67, 105–107). Therefore, MgtC decreases ATP amounts when the cytoplasmic Mg^2+^ concentration is low, which in turn decreases the transcription of rRNA genes, resulting in smaller amounts of ribosomes and a reduced rate of protein synthesis. In other words, *S*. Typhimurium adjusts the number of ribosomes to the Mg^2+^ amounts available for the assembly of functional ribosomes. Notably, while E. coli lacks an *mgtC* homolog, it also decreases rRNA synthesis in response to low cytoplasmic Mg^2+^ (67). Hence, E. coli and *S*. Typhimurium utilize different strategies to achieve the same goal, namely a reduction in ribosome numbers during Mg^2+^ limitation. In summary, bacterial species can control core biosynthetic activity in a like manner but often vary in how they implement feedback control in response to particular conditions.

### Bacterial growth, rather than metabolic activity, governs nonheritable resistance to antibiotics.

**FIG 3:**
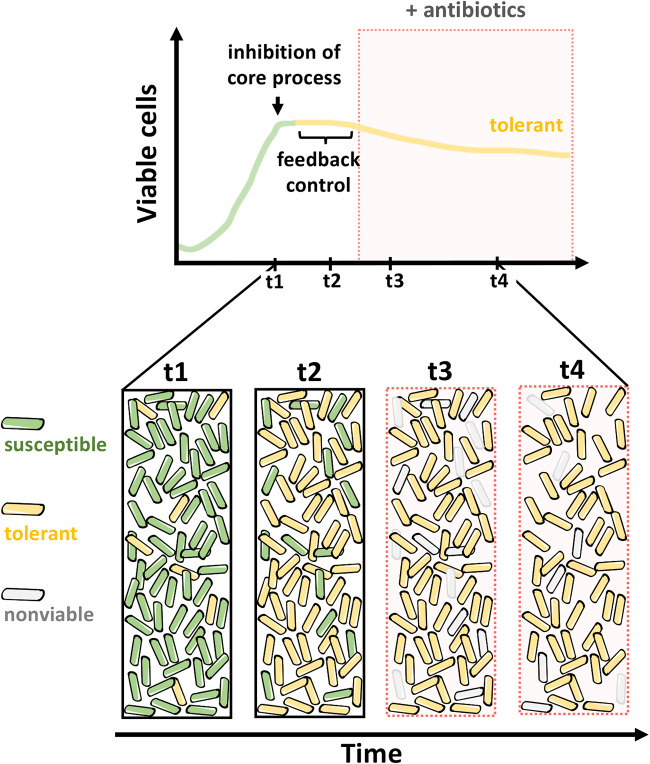
Schematic representation of antibiotic killing in a growing population of susceptible bacteria following inhibition of a core biosynthetic process (top). Feedback regulation promotes the inhibition of other major cellular processes, leading to multidrug tolerance. Cartoons depicting processes outlined in the top schematic (bottom).

Experiments conducted over the last 70 years have established a strong correlation between growth rate and antibiotic susceptibility, in that rapidly dividing cells are more prone to antibiotic-mediated killing (28, 35, 37, 38, 108–117). This correlation holds for different bacterial species and for antibiotics that target different core processes. Feedback control on core cellular processes, which determines the rate of biosynthetic activities targeted by antibiotics and dictates growth rate, is most likely responsible for this phenomenon (Fig. 3).

Surprisingly, it has been argued that susceptibility to antibiotics is directly related to ATP amounts, even in conditions of energy abundance—when a large fraction of cellular ATP does not participate in biosynthetic reactions fostering growth—and apparently, growth rate no longer correlates with the efficacy of antibiotic killing (103). These results are hard to reconcile with the large body of work demonstrating a strong correlation between growth rate and antibiotic susceptibility (28, 35, 37, 38, 108–117), especially because these studies were largely conducted under high-energy conditions (28, 108–114, 116).

It has been known for over 60 years that the chemical composition of cells changes when growth rate is modulated by nutrient availability (50, 76, 77). Therefore, it is puzzling to see a recent publication examine the effects of growth rate and ATP amounts on antibiotic susceptibility by altering the nutrient content of the growth medium (103). The problem with the chosen experimental strategy is that many physiological parameters are not controlled under disparate growth conditions. For instance, changes in nutrient availability may affect membrane permeability, ultimately altering antibiotic susceptibility (118, 119). In this context, the association between ATP levels and antibiotic susceptibility would simply be an epiphenomenon. Interestingly, in this work, ATP amounts are similar in bacteria experiencing energy abundance, whether grown at 25°C or 37°C, despite the growth rate increasing with temperature (103). However, modulation of growth rate by temperature does not alter the chemical composition of bacteria (50, 76, 77). Actually, in direct contradiction to the proposed model, it was reported that bacteria rapidly growing at 37°C are killed faster by antibiotics than their slow-growing counterparts at 25°C (103).

Finally, if ATP abundance determines the efficacy of killing independently of growth rate and type of antibiotic, then two non-mutually exclusive conclusions would follow: (i) high ATP abundance should increase the rate of all biosynthetic processes targeted by antibiotics—translation, cell wall biosynthesis, and DNA replication—but the increase in the activity of these processes would not result in a concomitant increase in biomass (i.e., growth). This means that an increase in ATP abundance would lead to an increase in rRNA, resulting in a higher number of ribosomes and, thus, higher translation rates, but that no extra proteins would be synthesized. Also, (ii) antibiotics should kill bacteria independently of the activity of their respective targets but dependently on the amount of ATP in the cells. That is, the rate of killing by bactericidal antibiotics would depend on something other than their effect on the activity of their cellular targets. The latter conclusion is reminiscent of the proposal that a wide variety of classes of antibiotics mediate killing by the generation of reactive oxygen species (120), a notion disputed by several independent groups (121–123).

### Conclusions.

In summary, we posit that there is no genetic program specifically devoted to nonheritable resistance to antibiotics. That is, a wide variety of growth and genetic conditions alter antibiotic resistance (124, 125). Therefore, a given gene or biochemical pathway may promote or inhibit resistance depending on the growth conditions. In this context, this hypothesis can explain both the inability of genetic screens to isolate mutations rendering cells unable to form persisters (126, 127) and the myriad of disruptive genetic and environmental contexts that lead to multidrug tolerance (8, 12, 13, 22, 24–29, 39–45).

We propose that the phenomenon of persister cell formation is the outcome of an evolved, essential property, namely feedback regulation of core cellular processes. Feedback regulation enables cells to buffer disturbances arising from external and internal perturbations that cause unbalanced growth and loss of viability. Typically, impairment of any major biosynthetic activity will lead to the inhibition of other vital cellular activities. Although a measurable outcome of this process is temporary immunity to multiple antibiotics, this is unlikely to be its evolved purpose.

Finally, the hypothesis presented above provides a conceptual framework that explains (128–130) and predicts that the disruption of feedback regulation should eradicate persisters. That is, persisters should be eliminated by imposing conditions that irreversibly abolish the core biosynthetic machinery when inactive core cellular components are degraded or the cytoplasmic environment is disrupted during a lethal antibiotic exposure (128–130). The identification of compounds that accomplish these tasks will result in the elimination of antibiotic persisters.
